# A walk on the wild side: A review of the epidemiology of *Toxocara canis* and *Toxocara cati* in wild hosts

**DOI:** 10.1016/j.ijppaw.2023.10.008

**Published:** 2023-10-26

**Authors:** Celia V. Holland

**Affiliations:** Department of Zoology, School of Natural Sciences, Trinity College, Dublin 2, Ireland

**Keywords:** *Toxocara canis*, *Toxocara cati*, Wild hosts, Canid, Felid, Epidemiology

## Abstract

*Toxocara* species are cosmopolitan nematode parasites of companion, domestic and wild hosts. Of the 26 known species of *Toxocara,* only *Toxocara canis* and *Toxocara cati* are definitively zoonotic. The significance of wild carnivores as definitive hosts of *T*. *canis* and *T*. *cati* respectively, has received far less attention compared to domestic dogs and cats. Complex environmental changes have promoted increasing contact between wildlife, domestic animals and humans that can enhance the risk of pathogen spillover. This review lists a total of 19 species of wild canid host that have been shown to act as definitive hosts for *T*. *canis* and a total of 21 species of wild felid host. In general, the number of publications focusing on felid host species is fewer in number, reflecting the general paucity of data on *T*. *cati*. The wild canids that have received the most attention in the published literature include the red fox (*Vulpes vulpes*), the wolf (*Canis lupus)*, and the golden jackal (*Canis aureus*). The wild felid species that has received the most attention in the published literature is the Eurasian lynx (*Lynx lynx*). Some non-canid and non-felid hosts also act as definitive hosts of *Toxocara* species. Certainly, red foxes would appear to be the most significant wild species in terms of their potential to transmit *Toxocara* to domestic dogs and humans via environmental contamination. This can be explained by their increasing population densities, encroachment into urban areas and their dietary preferences for a wide range of potential paratenic hosts. However, a major challenge remains to assess the relative importance of wild hosts as contributors to environmental contamination with *Toxocara* ova. Furthermore, one major constraint to our understanding of the significance of wildlife parasitism is a lack of access to samples, particularly from rare host species.

## Highlights

• 19 species of wild canid host act as definitive hosts for *Toxocara canis*.
• 21 species of wild felid host act as definitive hosts for *Toxocara cati*.
• Canid species of importance: red fox (*Vulpes vulpes*), wolf (*Canis lupus*), golden jackal (*Canis aureus*).
• Felid species of importance: Eurasian lynx (*Lynx lynx*).
• Red fox is the host species with the most potential to transmit *Toxocara.*

## Graphical abstract

**Figure:**
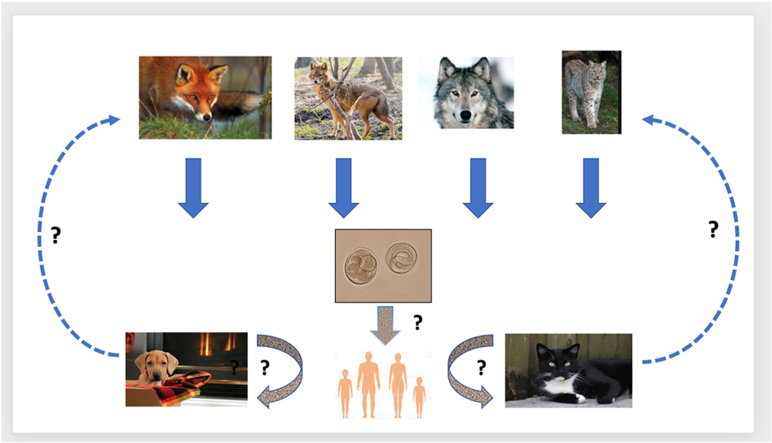

## 1 Introduction

*Toxocara* species are cosmopolitan nematode parasites that infect a wide range of companion, domestic and wild animals as definitive and paratenic hosts, via multiple routes of transmission, producing long-lived tissue-inhabiting larvae and resistant eggs that can survive in the external environment. Despite increasing awareness of their zoonotic potential and public health significance, gaps in our understanding of certain key aspects of the parasite's biology and epidemiology remain.

A recent review detailed 26 recognised species of the genus *Toxocara* with only *T*. *canis* and *T*. *cati* denoted to be zoonotic (Ziegler and Macpherson, 2019). The evidence that *T*. *cati* is a zoonosis is less well established compared to *T*. *canis* and this is further exacerbated by the fact that at present it is not possible to distinguish between exposure to the two species in humans using serology (Maciag et al., 2022). *T*. *canis* and *T*. *cati* are common and widespread nematode parasites of dogs (Rostami et al., 2020a) and cats (Rostami et al., 2020b) respectively, that act as definitive hosts, and release resistant eggs into the environment, resulting in extensive environmental contamination (Fakhri et al., 2018). The main routes of transmission to humans are through the ingestion of embryonated eggs from soil or on soil-contaminated hands, food or utensils (Ma et al., 2018). Other routes of transmission include the ingestion of organs from infected paratenic hosts, for example bovine liver (Yoshikawa et al., 2008). More recently, the presence of embryonated eggs on the hair of both dogs and cats has been implicated as a potential source of infection (Holland, 2017). However, based upon the available evidence, there is a low risk of transmission associated with the very low numbers of embryonated eggs found on hair (for example see Keegan and Holland, 2010), but the suitability of hair as a medium for oval development should not be ignored (Keegan and Holland, 2012). A recent global analysis estimated that the worldwide seroprevalence of *Toxocara* species in humans is currently 19%, with seroprevalence being highest in the African region (35%) and lowest in the Eastern Mediterranean region at 8.2% (Rostami et al., 2019).

The significance of wild carnivores as definitive hosts of *T*. *canis* and *T*. *cati* respectively, has received far less attention compared to domestic dogs and cats (see Bowman, 2020 and chapters therein). Complex environmental changes in recent times, including the fragmentation and destruction of natural habitats and increased urbanisation have promoted increasing contact between wildlife, domestic animals and humans that can enhance the risk of pathogen spillover (Deplazes et al., 2004; Duscher et al., 2015; Hassell et al., 2017; Faust et al., 2018).

Furthermore, we are still in the process of understanding some of the basic rules that determine the complex interactions between zoonotic parasites and their hosts, particularly in the light of changing environmental conditions (Mackenstedt et al., 2015). In the context of a one health approach, the inclusion of wildlife hosts is important especially as such hosts may play a role in maintaining the life cycles of parasites that are transmissible to domestic animals and humans (Waindock et al., 2021). One approach to assessing the potential for wildlife parasite pathogen spillover, particularly with respect to zoonotic parasites such as *T*. *canis* and *T*. *cati*, is to assess the extent of infection in canid and felid wildlife hosts, the key focus of this review.

However, some caveats should be added first to explain how the literature on *T*. *canis* and *T*. *cati* in wildlife was assessed. The main focus of the paper is upon wild definitive hosts that have been found to harbour either directly assessed adult worms or from which indirectly assessed eggs have been detected in host faeces. In addition, the role of wild paratenic hosts, in particular small rodents, is addressed briefly. Paratenic hosts are defined as hosts in which development does not occur, but may serve to bridge an ecological, or trophic gap in a parasite's life cycle (Bush et al., 2001). No studies based upon captive animals were included as these animals were not considered to be of epidemiological or public health significance. Nor does the review encompass feral hosts such as feral dogs and cats. The table in supplementary materials (Table S1), details the diagnostic methods utilised for each study that was included. Out of a total of 169 studies, the vast majority used morphology of either adult worms or parasite eggs to determine the species of *Toxocara*. Only 7 studies (4.3%) employed molecular confirmation – two studies using *T*. *canis* worms (Lesniak et al., 2017; Vega et al., 2018), two *T*. *cati* worms (Heddergott et al; Vega et al., 2018) and two *T*. *cati* eggs (Peng *et*. *al*. 2020; Segeritz et al., 2021) and one *T*. *canis* eggs (Myšková et al., 2019). Recent molecular diagnostic approaches include those of Knapp et al. (2016) who developed a rapid sensitive one step quantitative PCR (qPCR) that allows the detection of *Toxocara* spp. in faecal material and a host faecal test derived from red fox, dog and cat material. The authors emphasised the importance of confirmation of carnivore faeces in order to avoid bias in field studies. The importance of developing molecular techniques to accurately diagnose ascarids in wild canids and felids has been advocated by Xie et al. (2020). These authors utilised *Toxocara* worms from captive wild carnivore hosts including red and arctic foxes, and jungle, leopard and Asian golden cats. Some authors have highlighted the methodological limitations of detecting *Toxocara* spp. eggs in the faeces (or scats) of wildlife hosts, as opposed to adult worms at necroscopy, including an often underestimation of parasite diversity, owing to intermittent egg shedding, biases towards hermaphroditic or female parasites, and limitations towards species that excrete their eggs via the intestinal tract (Lesniak et al., 2017).

One major constraint to our understanding of the significance of wildlife parasitism is a lack of access to samples, particularly from rare host species, and this limitation emphasises the importance of collaboration between wildlife biologists involved in population conservation programmes and parasitologists (Otranto et al., 2015). For example, to my knowledge, only a single study has been published on the helminths of free-ranging wild lions (*Panthera leo*) in Tanzania. Analysis of faecal samples collected from 35 lions revealed a prevalence of 9% for *T*. *cati* (Bjork et al., 2000). In this instance, as in many others, indirect sampling via faecal samples was the only realistic and ethical option available.

## 2 Canidae as hosts for *T*. *canis*

*T*. *canis* is transmissible to wild canids as a result of ingestion of embryonated eggs from the environment, transplacental transmission of reactivated somatic larvae and the consumption of paratenic hosts mainly small rodents and birds (Otranto and Deplazes, 2019), although the relative importance of such routes will obviously vary by host species.

A total of 19 species of canids have been shown to act as definitive hosts for *T*. *canis* (see Table 1 and Table S1) with one species, the crab-eating fox, described as harbouring eggs identified only to the generic level as *Toxocara* spp. On the basis of the numbers of papers listed in Table S1 (Supplementary material), the top three species are described in more detail in the text and in Table 2, Table 3, Table 4 the Red fox (*Vulpes vulpes*), the European wolf (*Canis lupus*) and the golden jackal (*Canis aureus)*. The raccoon dog (*Nyctereutes procynoides)* has also be included as an example of an invasive canid species.

**Table 1:** Wild canid definitive hosts of *Toxocara canis*.

Common name	Latin name	*Toxocara* spp.	Stage detected
Red fox	*Vulpes vulpes*	*T*. *canis*	worms & eggs
Wolf	*Canis lupus*	*T*. *canis*	worms & eggs
Golden jackal	*Canis aureus*	*T*. *canis*	worms & eggs
Coyote	*Canis latrans*	*T*. *canis*	worms & eggs
Racoon dog	*Nyctereutes procynoides*	*T*. *canis*	worms & eggs
Arctic fox	*Vulpes lagopus*	*T*. *canis*	worms & eggs
Swift fox	*Vulpes velox*	*T*. *canis*	worms & eggs
Iberian wolf	*Canis lupus signatues*	*T*. *canis*	eggs
Darwin's fox	*Lycalopex fulvipes*	*T*. *canis*	eggs
Italian wolf	*Canis lupus italicus*	*T*. *canis*	eggs
Japanese red fox	*Vulpes vulpes japonica*	*T*. *canis*	worms
Kit fox	*Vulpes macrotis*	*T*. *canis*	worms
Andean fox	*Lycalopex culpaeus*	*T*. *canis*	worms
Pampas fox	*Lycalopex gymnocercus*	*T*. *canis*	worms
Crab eating fox	*Cerdocyonthous cerdocyon*	*Toxocara* spp	eggs
Gray fox	*Urocyon cinereoargenteus*	*T*. *canis*	eggs
Ethiopian wolf	*Canis simensis*	*T*. *canis*	eggs
Bush dog	*Speothus venaticus*	*T*. *canis*	eggs
African wild dog	*Lycaon pictus*	*T*. *canis*	eggs

**Table 2:** Studies on *Toxocara canis* in red foxes (*Vulpes vulpes*).

Location	Sample size	% prevalence	Abundance; intensity; range; k	Author & date
Australia^a^	1320	42.30%	5.9 (A)	Coman (1973)
	147	14.90%	7.7 ± 7.84 (I); 1-34	Dybing et al., (2013)
Austria	307	42.90%		Suchentrunk and Sattmann (1994)
Belarus	94	25.50%	range 2–40	Shimalov and Shimalov (2003)
Belgium	134	18.00%		Brochier et al., (2007)
Bulgaria	243	49.79%		Jancev and Ridjakov (1977)
Canada	61	71%		Smith (1978)
	271	25%		Wapenaar et al., (2013)
	155	11%		Bouchard et al., (2021)
Czech Republic	40	37.50%	3 (I); 1-24	Jankovska et al., (2016)
Croatia	85	28.23%	2.15 GM (I); 0-15	Rajkovic-Janje et al., (2002)
Denmark	68	23.50%	17.1 (A); 0-55	Willingham et al., (1996)
	1040	59.40%	4.1 (A)	Saeed et al., (2006); Saeed and Kapel (2006)
			7 (I); 1-116	
			k = 0.233	
	384	60.90%	2.6 (A)	Al-Sabi et al., (2013)
			4.3 (I); 1-28	
Estonia	108	29.60%	5 (I); 1-20	Laurimaa et al., (2016)
	131	13.70%		Tull et al., (2022)
Egypt	41	48.80%	2.3 ± 3.9 (A)	Radwan et al., (2009)
			5.4 ± 4.3 (I)	
France	154	51.30%		DeBlock et al., (1988)
Germany	3138	31.30%		Loos-Frank and Zeyhle (1982)
	80	43.80%		Waindock et al., (2021)
Greece	314	28.60%		Papadopoulus et al., (1997)
Iran	22	4.54%		Dalimi et al., (2006)
	84	42.70%		Zare-Bidak et al., (2010)
	62	27.41%		Razmjoo et al., (2014)
Ireland	77	37.70%	2.43 (A); 0-32	Wolfe et al., (2001)
	87	61%	4 ± 8 (A); 0-54	Roddie et al., (2008)
	91	19%		Stuart et al., (2013)
Italy	645	54.42%	5.05 ± 11.89 (A)	Di Cerbo et al., (2008)
			9.27 ± 14.85 (I); 0-162	
			k = 0.235	
	129	9.10%	0.5 (A)	Magi et al., (2009)
			6.1 (I); 1-20	
	57	52.60%	3.2 (A)	Fiocchi et al., (2016)
			6 (I); 1-30	
			VMR 13.1	
Japan	7	71%	Range 1-10	Sato et al., (1999)^b^
Kyrgyzstan	151	30.40%	2.01 (A)	Ziadinova et al., (2010)
			k = 0.30	
Lithunia	269	40.50%		Bruzinskaite-Scmidhalter et al., (2012)
Poland	1909	39.80%		Balicka-Ramisz et al., (2003)
	22	13.60%		Gorski et al., (2006)
	620	30.20%	12.1 (I); 1-84	Tylkowska et al., (2021)
Netherlands	139	73.70%	0–106	Borgsteede (1984)
	136	61%		Franssen et al., (2014)
Portugal	28	21.43%		Figueiredo et al., (2016)
Serbia	172	49.41%		Ilic et al., (2016)
	223	16.60%	0.3 (A)	Miljevic et al., (2019)
			1.7 (I); 1-6	
Slovak Republic	302	25.82%		Letková et al., (2006)
	1198	12.50%		Miterpakova et al., (2009)
Slovenia	428	38.30%		Rataj Vegles et al., (2013)
Spain	201	23%		Alvarez et al., (1995)
	67	4.40%		Criado-Fornelio et al., (2000)
	55	45.50%	4.9 ± 8.5 (I); 1-35	Martinez-Carrasco et al., (2007)
Switzerland	228	44.30%		Reperant et al., (2007)
	1481	12.10%		Koller et al., (2019)
				
Turkey	103	14.56%	0.52 (A)	Erol et al., (2021)
			3.6 (I); 1-9	
United Kingdom	280	63%	4.5 (A); 1-29	Hackett and Walters (1980)
	843	55.90%	4.01 ± 0.31 (A)	Richards et al., (1995)
			7.17 (I)	
			k = 0.288; VMR 20.45	
	588	61.60%	6.1 (I) (1–103)	Smith et al., (2003)
Yugoslavia	459	50.32%	range 1–27	Pavlovic et al., (1997)

a Invasive species.

b Japanese red fox - *Vulpes vulpes japonica*.

**Table 3:** Studies on *Toxocara canis* in wolves (*Canis lupus*).

Location	Sample size	% prevalence	Abundance; intensity; range; k	Author & date
Belarus	52	21.20%	range 1–12	Shimalov and Shimalov (2000)
Canada	98	2.04%	range 2–3	Holmes and Podesta (1968)
	601	0.30%		Stronen et al., (2011)
Croatia	400	2.80%		Hermosilla et al., (2017)
Egypt	43	62.80%	8.09 ± 12.5 (A)	Radwan et al., (2009)
			15.1 ± 13.7 (I); 1-61	
Estonia	26	8%		Moks et al., (2006)
Ethiopia^a^	94	14.90%	Median epg 3 (2–3.75 IQ) Max epg 9	van Kesteren et al., (2014)
Kazakhstan	41	39%	5.52 (A)	Abdybekova and Torgerson (2012)
Germany	53	11%		Lesniak et al., (2017)
	69	13.04%		Bindke et al., (2019)
Greece	6	16.60%		Papadopoulus et al., (1997)
Italy	89	17%	1.93 ± 1.12 (I); 1-9	Guberti et al., (1993)
	3	33%	0.3 (A)	Fiocchi et al., (2016)^b^
			1 (I); range 1	
	37	4.34%		Paoletti et al., (2017)^c^
	42	9.50%	2.7 (I); 1-6	de Macedo et al., (2019)
Latvia	34	5.80%	0.05 ± 0.04 (I)	Balgrade et al., (2003)
Poland	52	13.50%	2.04 ± 1.23 (A GM)	Kloch et al., (2005)
	89	5.60%		Popiołek et al., (2007)
	58	3.5%%		Szczesna et al., (2007)
	72	6.90%		Borecka et al., (2013)
Portugal	11	9.09%		Figueiredo et al., (2016)^d^
Serbia	102	3.90%	range 4–19	Ćirovic et al., (2015)
				
Slovakia	256	3.10%		Čabanová et al., (2017)
Spain	18	5.50%		Dominguez and de la Torre (2002)^e^
	47	6.40%	3.3 ± 2.2 (I)	Segovia et al., (2003)
	50	6%	0.20 ± 0.15 (A)	Segovia et al., (2003)
			range 1–7	
			VMR = 5.3	
	93	10.75%		Munoz et al., (2018)^f^
USA	204	0.98%		Byman et al., (1977)

a Ethiopian wolf; *Canis simensis*; Eggs; designated as *Toxocara* spp.

b Italian wolf; *Canis lupus italicus*.

c Apennine wolf; *Canis lupus italicus*.

d Iberian wolf; *Canis lupus signatus*.

e Iberian wolf; *Canis lupus signatus*.

f Iberian wolf; *Canis lupus signatus*.

**Table 4:** Studies on *Toxocara canis* in the golden jackal (*Canis aureus*).

Location	Sample size	% prevalence	Abundance;	Author & date
			intensity; range	
Estonia	65	4.60%		Tull et al., (2022)
Greece	5	40%		Papadopoulus et al., (1997)
Hungary	20	20%		Takacs et al., (2014)
India	879	22.86%		Sheikh et al., (2023)
Iran	20	10%		Sadighian (1969)
	10	10%		Dalimi et al., (2006)
	79	5%		Meshgi et al., (2009)
	56	12.50%		Razmjoo et al., (2014)
	11	27.20%		Eslahi et al., (2017)
	43	4.50%		Siyadatpanah et al., (2019)
Serbia	447	1.60%	7.86 ± 2.14 (I)	Ćirovic et al., (2015)
	60	23.30%		Ilic et al., (2016)
	64	4.70%	0.2 (A)	Miljevic et al., (2019)
			4.3 (I); 1-11	
Switzerland	4	25%		Frey et al., (2022)
Tunisia	31	16%	3.2 (I)	Lahmar et al., (2014)

### 2.1 Red fox (*Vulpes vulpes*)

A total of 10 species of fox are listed in Table 1 as hosts of *T*. *canis* detected by either eggs, worms or both. This group of hosts are, therefore, the most frequently listed in Table S1. These are Red fox (*Vulpes vulpes*), Arctic fox (*Vulpes lagopus*) (Myšková et al., 2019), Swift fox (*Vulpes velox*) (Criffield et al., 2009), Darwin's fox (*Lycalopex fulvipes*) (Jiménez et al., 2012), Japanese red fox (*Vulpes vulpes japonica*) (Sato et al., 1999), Kit fox (*Vulpes macrotis) (*Ubelaker et al., 2014)*,* Andean fox (*Lycalopex culpaeus) (*Vega et al., 2021)*,* Pampas fox (*Lycalopex gymnocercus) (*Moleon et al., 2015), Crab-eating fox (*Cerdocyonthous cerdocyon) (*Santos et al., 2012) and Gray fox (*Urocyon cinereoargenteus) (*Hernández-Camacho et al., 2011).

As a definitive host of *T*. *canis*, the red fox has received the most attention in the published literature. Red foxes are the most widespread wild carnivores in the world. They are highly adaptable to a range of habitats due to their opportunistic feeding behaviour and are found in almost all regions of the northern hemisphere (Hoffmann and Sillero-Zubiri, 2016). Red fox density has increased in many European countries, a trend that is attributable principally to the impact of a successful rabies vaccination campaign of wild animals, an increase in anthropogenic food sources, and the impact of environmental factors (Gloor et al., 2001).

Factors that influence the importance of red foxes as sources of pathogen spillover, include the increased population density of foxes, their susceptibility to pathogens that can also infect domestic animals and humans (such as *T*. *canis*), their hunting preference for small mammals and their wide distribution and close proximity to human settlements (Duscher et al., 2015). In peri-urban and urban areas of Estonia, Plumer et al. (2014) detailed how contact between red foxes, pets and humans has changed from sporadic to constant, thereby enhancing the risk of successful parasite transmission. Otranto et al. (2015) have emphasised how the predominance of small mammals and birds in the diet of red foxes, all of which may act as paratenic hosts for *Toxocara* spp., may impact upon the epidemiology of *T*. *canis*.

Publications from 31 countries, published between 1973 and 2022, are listed in Table 2 and full details of each paper surveyed are included in Table S1. Unlike some other wild hosts, prevalences in red foxes have fluctuated quite considerably with values as low as 4% in Spain (Criado-Fornelio et al., 2000) (but with two other studies recording prevalences of 23% and 45% respectively) to as high as 74% in the Netherlands (Borgsteede, 1984). However, the majority of studies record moderate to high prevalences. Even within a single country, prevalences can fluctuate quite markedly. For example, three Canadian studies recorded prevalences of 11, 25 and 70% respectively. In terms of abundance, an epidemiologically more robust measure of helminth population dynamics, figures rarely exceed an average of 10 with the exception of studies by Willingham et al. (1996) from Denmark (17.1) and a Polish study by Tylkowska et al. (2021) (12.1). Ranges and values of k demonstrate aggregation with several concordant values of 0.2 (see Table 2).

A number of studies have compared the efficacy of detection using egg counts versus worm counts and not surprisingly, have demonstrated the reduced efficacy of egg counts as a measure of prevalence. For example, Martinez-Carrasco et al. (2007) recorded prevalences of 12.5% by egg count versus 45.5% by worm count and Saeed and Kapel (2006) recorded prevalence based on eggs as 41%, but that based on worms as 76%. In a recent comprehensive study, Marchiori et al. (2023) compared copromiscroscopy utilising a classical flotation technique (FT) using a zinc chloride solution to a scraping filtration and counting technique (SFCT) in 150 red foxes from Poland. Both *T*. *canis* and *Toxascaris leonina* were combined as ascarids in their analysis. Concordance values were 62.7% and sensitivity was 36.3% but increased to 46.1% after the exclusion of single-sex infections and infections solely by immature worms.

In a novel approach based on a large sample of Polish red foxes, Tylkowska et al. (2021) divided the intestine into three sections and found a statistically significantly lower prevalence in the ileum (10.3%) versus the duodenum (20.7%) and the jejunum (26%) based upon worm counts at autopsy.

Several studies have provided particularly comprehensive data sets on the epidemiology of *T*. *canis* in red foxes. Richards et al. (1993) recorded higher prevalences of *T*. *canis* among male versus female red foxes and juvenile versus adult red foxes. Aggregation was pronounced with k values of 0.328 in males and 0.223 in females and variance to mean ratios of 16.03 and 14.26 respectively. In Denmark, Saeed et al., (2006) recorded a high prevalence of 59% among over 1000 red foxes. Mirroring the results of Richards and colleagues, prevalence and abundance of *T*. *canis* was higher in male versus females red foxes and in cubs versus older animals. In addition, *T*. *canis* was more prevalent and abundant in red foxes sampled from rural versus urban areas. In a very detailed study that included an analysis of worm fecundity, Saeed and Kapel, 2006 revealed that female worm fecundity was lower in female compared to male red foxes, higher in cubs versus young and adults and higher in summer compared to other seasons. In an extensive study of over 600 red foxes from Northern Italy, Di Cerbo et al. (2008) explored the contribution of environmental variables and the structure of the red fox population on the composition of the intestinal helminth community. With a prevalence of 54.4% and a mean abundance of 9.3 worms, *T*. *canis* was a dominant parasite that was geographically widespread and abundant. Prevalence and abundance of *T*. *canis* demonstrated high spatial variability with a peak prevalence and abundance of 71% and 10.4 respectively in the Aosta region.

In Switzerland, Koller et al. (2019) employed a standardised sampling scheme based on the collection of red fox faecal samples along transects in 1-km^2^ grid cells dispersed over the whole country and found that the prevalence of *T*. *canis* was significantly lower south of the Alps. The authors advocated the use of such a standardized method, that allows for follow-up surveys of red fox scats without culling interventions, a useful procedure for assessing and monitoring on a large scale and over time the infection pressure on dog populations.

In Australia, the red fox is an invasive species (Bezerra-Santos et al., 2023) that was introduced for sport and to control rabbits (Mackenstedt et al., 2015). Australian red foxes are now known to be infected with *T*. *canis* as shown by Dybing et al. (2013) who recorded a prevalence of 15% in 147 foxes sampled. In contrast to many other parts of the world, *T*. *canis* in domestic dogs is more effectively controlled in Australia (Thompson, 2023) and environmental contamination is low (see Massetti et al., 2022). The red fox is now considered to be a potentially important reservoir that may contribute to spillover of infection to both domestic dogs and humans (Bezerra-Santos et al., 2023).

To conclude, the wide distribution of red foxes coupled with their ecological plasticity enabling them to live successfully in a variety of contrasting environments, enhances their importance as potential transmitters of *T*. *canis* to both domestic dogs and humans. Clearly further studies are needed, that incorporate the one health approach, in order to successfully implement surveillance and risk assessment of wildlife parasite spillover (Waindock et al., 2021).

### 2.2 Wolf *(Canis lupus)*

Several species of wolves have been the focus of helminth parasite surveys including *Canis lupus* (Linnaeus, 1758), the European wolf, the Italian or Apennine wolf – *Canis lupus italicus*, a subspecies of the gray wolf native to the Italian Peninsula, the Iberian wolf *Canis lupus signatus,* a subspecies living in the Iberian peninsula and the Ethiopian wolf – *Canis simensis* which is endemic to the Ethiopian highlands. *C*. *simensis* is the world's rarest canid with its main prey being the endemic giant mole rat (Sillero-Zubiri and Gottelli, 1995). To my knowledge, the only published study on the Ethiopian wolf is by van Kesteren et al. (2014) who recorded a prevalence of *T*. *canis* of 15% with a very low median epg of 3.

The extent of *T*. *canis* infections in European wolves (*Canis lupus*) have received some attention in the published literature, although to a much lesser extent than that given to red foxes, with studies from 15 different countries (Table 3). The first and most obvious observation is the much lower prevalences of *T*. *canis* in wolves, compared to red foxes, with many values below 10%. Notable exceptions are the high prevalences reported from Egypt (Radwan et al., 2009) and Kazakhstan (Abdybekova and Torgerson 2012). Of interest, is the fluctuation in prevalences between four studies from Italy ranging from 4.34% to 33% (albeit based upon a sample of 3). In general, worm burdens are low with only one study including a measure of aggregation (Segovia et al., 2003).

The prevalence of *T*. *canis* in wolves varies quite considerably (Table 3) and this may be explained in part, by the fact that wolves rarely consume small mammals compared to other canids (Guberti et al., 1993). In Latvia, a study of the feeding habits of wolves revealed wild ungulates (cervids and wild boar) and beavers as the wolves’ most frequent prey. Several species of small and medium-sized carnivores including the domestic dog, raccoon dog, red fox, badger, otter and weasel as well as some small rodents and insectivores were detected in the stomach content of wolves. However, their occurrence was comparatively low (Andersone and Ozolins, 2004). The range of parasite species infecting wolves may vary markedly dependent upon locally available prey species, the wolf biome and zoogeographical region, reflecting prey species biodiversity, the particular wolf-infective parasite stages they harbour and their relative population densities (Craig and Craig 2005; Guberti et al., 1993). An extensive study by Ståhlberg et al., (2017) compared the feeding habits of European wolves (*Canis lupus*) from Northern and Southern Europe exemplified by Sweden and Italy. Diet composition varied between the two locations with higher prey abundance and choice associated with the greater ecological diversity of Italy compared to Sweden. However, in both locations, small mammals played a very small part in wolf diets in contrast to large prey such as moose in Sweden and wild boar and roe deer in Italy.

Differences in the prevalence of *Toxocara* between studies may be explained in part by the diagnostic method employed and environmental conditions at collection. Popiołek et al. (2007) highlighted how data from wolf scats based upon coprological analysis, including their own data at 5.6%, tend to be lower (see Table 3) than those values based on necropsies that are usually much higher, for example 17% in Italy, 16.6% in Greece or 21.2% in Belarus (Guberti et al., 1993; Papadopoulus et al., 1997; Shimalov and Shimalov, 2000). According to Kloch and Bajer (2003) this may suggest that the eggs could be washed out of the faeces before collection, for example by rain or thawing snow.

Daszak et al. (2000) has emphasised the potential for pathogen transfer between wolves, companion animals, livestock and people, especially in anthropogenically modified cultural landscapes with a high human population density (Chapron et al., 2014). In a study that focused upon the recolonisation of wolves in Germany and the consequent founder effects on endoparasite diversity, LesniakHeckmann et al. (2017) suggested that it might be useful to implement endoparasite screening in order to benefit both domestic dog owners and hunters in wolf habitats, and to provide information to assist in well-informed decisions on anthelminthic dog treatment.

### 2.3 Golden jackal *(Canis aureus)*

Of the other canids listed in Table 1, the golden jackal has received some attention including a synoptic overview of its parasites (Gherman and Mihalca, 2017). This carnivore is a wild canid with one of the widest distributions in the world, inhabiting Europe, the Middle East and Central and Southeast Asia (Moehlman and Hayssen, 2018). Recently, the species has been experiencing a rapid large-scale expansion almost all over Europe (Spassov and Acosta-Pankov, 2019). Studies from 8 different countries have revealed prevalences of *T*. *canis* that can exceed those of wolves but generally do not reach some of the higher values observed in foxes (Table 4). As hosts of helminth parasites, jackals have been of particular focus in Iran and demonstrate prevalences ranging from as low as 4.5%–27.2%, albeit in a sample size of just 11 animals. Overall, there is a lack of sufficient data on the abundance and intensity of *T*. *canis* in jackals.

In a recent review of the golden jackal's dietary composition, this canid was found to eat mainly small mammals (54% biomass), followed by domestic animals, ungulates and plants (Lange et al., 2021). Their diet also includes birds and lagomorphs, but dietary composition varies considerably by season and geographic location. The authors concluded that the golden jackal can be described as a highly adaptive opportunistic omnivore (Lange et al., 2021). The high consumption of small rodents coupled with birds, and in some instances even invertebrates, is likely to increase their exposure to parasites (Gherman and Mihalca, 2017) particularly *Toxocara* and could explain the prevalences observed in some of the studies listed in Table 4. In a recent paper that explored the helminth parasites of golden jackals in Estonia (a new mammal species in that location), Tull et al. (2022) emphasised the potential significance of jackals as vectors of zoonotic helminths. A potential hazard to public health was identified since the habitats of the red fox and golden jackal overlap to some extent with that of free-ranging dogs, and with humans who interact with infected dogs. Furthermore, resettlement of key host species such as wolves and jackals in Europe may create new patterns of zoonotic infection, but the risk to human health is likely to be low due to small population sizes of these carnivores (Otranto and Deplazes, 2019).

### 2.4 Raccoon dog (*Nyctereutes procyonoides*)

The raccoon dog (*Nyctereutes procyonoides*), is an invasive species in Europe and is well established in Northern, Eastern and Central Europe (Laurimaa et al., 2016). This Estonian study identified 17 helminth species among a sample of 255 raccoon dogs and concluded that given the increase in both the numbers and range of such hosts in Europe, this host species represents a potentially significant source of environmental contamination with zoonotic parasites. However, Otranto and Deplazes (2019) highlighted that raccoon dogs tend to concentrate their faeces in confined areas (latrines) (Yamamoto and Hidaka, 1984) which may restrict more widespread environmental contamination with *Toxocara* eggs. Three comparative studies from Lithuania, Denmark and Germany have revealed that the prevalence of *T*. *canis* is lower in raccoon dogs compared to foxes [in foxes 60.90% versus 13.10% (Al-Sabi et al., 2013), 40.50% versus 17.6% (Bruzinskaite-Schmidhalter et al., 2012) and 43.80% versus 33.0% (Waindock et al., 2021)]. However, it should be noted that in the most recent study by Waindok et al. the two prevalence values were reported to be much more similar. The relative abundances in Danish foxes were 2.6 (range 1–28) versus 0.2 (range 1–8) in raccoon dogs (Al-Sabi et al., 2013).

An analysis of a large data set of mammalian host-parasite associations found that the wildlife species with the highest number of zoonotic helminth parasites were the red fox, (*Vulpes vulpes*), the European wolf (*Canis lupus*) and the raccoon dog (*Nyctereutes procyonoides*) (Wells et al., 2018).

## 3 Felidae as hosts of *T*. *cati*

*T*. *cati* is transmissible to wild felids as a result of ingestion of embryonated eggs from the environment and the consumption of paratenic hosts mainly small rodents and birds (Otranto and Deplazes, 2019). Domestic cats have been shown to also transmit *T*. *cati* via the transmammary route (Coati et al., 2004) but such a route of transmission has not been confirmed in wild felids (Otranto and Deplazes, 2019).

A total of 21 species of felids have been shown to act as definitive hosts for *T*. *cati* (see Table 5 and Table S1) with one species, the Southern tiger cat (*Leopardus guttulus)* described as harbouring eggs detected only to the generic level as *Toxocara* spp. In general, the number of publications focusing on felid host species is fewer in number, reflecting the general paucity of data on *T*. *cati* (Maciag et al., 2022). On the basis of the numbers of papers listed in Table S1 (Supplementary material), the top two species, Eurasian lynx (*Lynx lynx*) and Bobcat (*Lynx rufus)* are described in more detail in the text and in Table 6. The other two Lynx species – Iberian lynx (*Lynx pardinus*) and Canadian lynx (*Felis canadensis*) are also included for completeness.

**Table 5:** Wild felid definitive hosts of *Toxocara cati*.

Common name	Latin name	Toxocara spp.	Stage detected
Bobcat	*Lynx rufus/Felis rufus*	*T*. *cati*	worms & eggs
Eurasian lynx	*Lynx lynx*	*T*. *cati*	worms & eggs
Puma/Cougar	*Felis concolor/Puma concolor*	*T*. *cati*	worms & eggs
Kodkod/Guigna	*Leopardus guigna/Oncifelis guigna*	*T*. *cati*	worms & eggs
Geoffrey's cat	*Leopardus geoffroyi/Oncifelis geoffroyi*	*T*. *cati*	worms
Iberian lynx	*Lynx pardinus*	*T*. *cati*	worms & eggs
Canadian lynx	*Felis canadensis*	*T*. *cati*	worms
Florida panther	*Felis concolor corgi/Felis concolor coryi/Puma concolor coryi*	*T*. *cati*	worms & eggs
Amur/Siberian tiger	*Panthera tigris altaica*	*T*. *cati*	eggs
Leopard	*Panthera pardus*	*T*. *cati*	eggs
Persian leopard	*Panthera pardus saxicolor*	*T*. *cati*	eggs
	*Panthera pardus kotiya*		
Ocelot	*Leopardus pardalis*	*T*. *cati*	eggs
Tsushima leopard cat	*Felis bengalensis euptilurus*	*T*. *cati*	worms
Iriomote cat	*Felis iriomotensis*	*T*. *cati*	worms
Fishing cat	*Prionailurus viverrinus*	*T*. *cati*	eggs
Tiger	*Panthera tigris*	*T*. *cati*	eggs
Jaguar	*Panthera onca*	*T*. *cati*	eggs
Lion	*Panthera leo*	*T*. *cati*	eggs
Southern tiger cat	*Leopardus guttulus*	*Toxocara* spp.	eggs
Pallas' cat	*Otocolobus manul*	*T*. *cati*	worms
European wildcat	*Felis silvestris*	*T*. *cati*	worms & eggs

**Table 6:** Studies on *Toxocara cati* in *Lynx* species.

Species	Location	Sample size	% prevalence	Abundance; intensity; range	Author & date
Bobcat					
*Lynx rufus/*	Mexico	30	80%		Camacho-Macías et al., (2018)
*Felis rufus*					
	USA	50	42%		Rollings (1945)
		16	75%		Miller and Harkema (1968)^a^
		66	17%	5 (I); 1-27	Stone and Pence (1978)^b^
		143	89%	44 (I); 1-1258	Watson et al., (1981)^c^
		10	80%	13 (I); 1-39	Watson et al., (1981)^d^
		75	39%	3 (I); 1-13	Tiekotter (1985)
		8	37.50%		Heidt et al., (1998)
		43	23.25%	4 (A)	Hiestand et al., (2014)
				14 (I)	
		67	25.00%	3 ± 11 (A)	Hiestand et al., (2014)
				14 ± 18 (I); 1-60	
		103	4.85%		Carver et al., (2012)
Eurasian lynx					
*Lynx lynx*	Estonia	37	68%	17.1 (I); 2-78	Valdmann et al., (2004)
	Finland	339	71%		Deksne et al., (2013)
		296	93%	16 (median I); 1-167	
		2756*	84%	18 ± 25.5 (A)	Virta et al., (2022)
				21.2 ± 26 (I); 1-218	
				k = 0.513	
	Germany	24	46%		Segeritz et al., (2021)
	Latvia	42	77%	12.8 (I); 1-52	Balgrade et al., 2003
	Poland	7	15%		Gorski et al., (2006)
		100	26%	6.46 ± 1.9 (mean epg); 1-48	Szczesna et al., (2008)
		11	64%	17 (A)	Kolodziej-Sobocinska et al., (2018)
				26 (I)	
	Switzerland	58	40%		Schmidt-Posthaus et al., ( 2002)^e^
Iberian lynx					
*Lynx pardinus*	Spain	8	38%	5.3 (I); 1-11	Torres et al., (1998)
		5	20%%	0.20 ± 0.44 (A)	Millan and Casanova (2007)
		32	16%	4566.7 (mean epg)	Figueiredo et al., (2021)
				100-11,050	
Canadian lynx					
*Felis canadensis*	Canada	113	4%		van Zyll de Jong (1966)
		274	33%	3.9 (I); 1-20	Smith et al., (1986)

a *T. mystax*.

b *T. mystax*.

c West Virginia; *T. mystax*.

d Georgia; *T. mystax*.

e Designated *Toxocara* spp.

### 3.1 Eurasian lynx *(Lynx lynx)*

The felid species that has received the most attention in the published literature is the Eurasian lynx (*Lynx lynx*). There are four species of the genus Lynx Kerr, 1792: two in America, *L*. *canadensis* Kerr, 1792 and *L*. *rufus* (Schreber, 1777), one Euro-Asiatic, *L*. *lynx* (Linnaeus, 1758), and one species endemic to the Iberian Peninsula, *L*. *pardinus* (Temminck, 1827; Wilson and Reeder, 1993). The Iberian lynx is considered to be one of the most threatened Felidae species in the world (Figueiredo et al., 2021) and only three studies on *T*. *cati* in this species are described in Table 6.

Studies on *L*. *lynx* from 7 countries have revealed that most prevalence values of *T*. *cati* are high compared with other *Lynx* species and particularly so in Finland (Table 6). Average abundance values are also high with mean values of 18 worms/host in Finland and 17 in Estonia and Poland respectively. Data on the diet of lynx from Poland have identified rodents as a common prey item (Schmidt, 2008).

The history of the Eurasian lynx population in Finland has provided an opportunity to study the potential effects of a dramatic population increase and expansion of a solitary apex predator on their parasite prevalence and abundance. The extensive study of Finnish lynx by Virta et al. (2022) that was conducted between 1999 and 2015, reported worm burdens that ranged from 1 to 218 worms/host with a k value of 0.513 indicating significant aggregation. This study concluded that despite considerable increases in the lynx population over the period of the study, *T*. *cati* prevalence did not vary significantly between years nor by lynx density, and that abundance was not dependent on lynx population density either. The authors acknowledge the lack of information concerning the extent of environmental contamination and the level of *T*. *cati* infection in paratenic hosts in Finland as explanatory variables, while highlighting the potential contribution from domestic cats (see for example Näreaho et al., 2012). Importantly, they emphasise that the role of free-ranging wildlife species in the maintenance of zoonotic pathogens should not be forgotten in One Health investigations.

### 3.2 Bobcat *(Lynx rufus)*

The bobcat, *L*. *rufus* or *Felis rufus* is the most widely distributed carnivore in North America (Roberts and Crimmins, 2010) with a tolerance for fragmented habitats (Riley et al., 2004). The majority of studies on *T*. *cati* in bobcats are from North America with the exception of one study from Mexico (Table 6). As was the case for the Eurasian lynx, bobcats also demonstrate high prevalences of *T*. *cati* although worm burdens tend to be more modest with the exception of the bobcats from West Virginia (Watson et al., 1981). In a novel approach, Hiestand et al. (2014) modelled the potential presence of *T*. *cati* in bobcats from Southern Illinois and found that all climatic variables were low contributors to model creation, whereas land surface cover was as an important variable for the presence of *T*. *cati*.

The diet of bobcats includes lagomorphs, rodents, white tailed deer, and birds as common prey species (Litvaitis et al., 1984; Toweill and Anthony, 1988). Stone and Pence (1978) highlight the predominantly carnivorous diet of bobcats with its diet in West Texas consisting of rodents (55% aggregate volume), rabbits (39%), and birds (6%), all potential paratenic hosts for *Toxocara* spp.

## 4 Potential impact of helminth parasites such as *Toxocara* spp. on wildlife health

There is a dearth of information on the potential impact of helminth parasites in general, and *Toxocara* spp. in particular, on wildlife hosts. In a review of anti-parasite treatment experiments in wildlife, as a means of assessing their impact, Pedersen and Fenton (2015) outlined a total of 51 such studies. Among a range of wild animals, only 3 focused upon canid or felid hosts and only one provided information on host health status (Foster et al., 2006). Both treated and untreated Florida panthers (*Puma concolor coryi*) infected with several helminth species (Trematodes, Cestodes and Nematodes) were compared and no effect of anthelmintic treatment was observed. In fact, at the time of death, most of the untreated animals were found to be in good nutritional condition. However, it should be noted that the prevalence and abundance of *T*. *mystax* was low (6% and 2 worms respectively).

In a wide ranging and comprehensive meta-analysis, Shanebeck et al. (2022) analysed the energetic costs of sub-lethal helminth parasites in mammals. These authors concluded that mammal energetic condition is significantly affected by sublethal helminth parasites across all major host groups including carnivores. However, they also highlighted methodological limitations and disparities in wildlife research that are likely to reduce the resolution of such effects and emphasise the need for further investigation as mirrored in the earlier findings of Pedersen and Fenton (2015).

## 5 Other non-canid or non-felid hosts

A number of other hosts that are neither canids or felids, have been found to harbour eggs or worms of *Toxocara* spp. (Table 7). Three host species, the Asian palm civet (*Paradoxus hermaphroditus*) (Sepelage and Rajakaruna 2020), spotted hyena (*Crocuta Crocuta*) (Engh et al*,*. 2003) and stone marten (also known as beech marten) (*Martes foina*) (Olmedo et al., 2018; Figueiredo et al., 2018) have been described as harbouring *Toxocara* spp. eggs whereas the Egyptian mongoose (*Herpestes ichneumon*) (Radwan et al., 2009) and the American mink (*Neovision vison) (*Tull et al., 2022) were described as harbouring *T*. *canis* worms or eggs. In contrast, the eggs of *T*. *cati* were detected in the pine marten (*Martes martes)* by Borecka et al. (2013) and Gorski et al. (2006).

**Table 7:** Non-canid or non-felid hosts of *Toxocara* spp.

Common name	Latin name	Family names	*Toxocara* spp.	Stage detected
Spotted hyena	*Crocuta*	Family Hyaenidae	*Toxocara* spp.	Eggs
Egyptian mongoose	*Herpestes ichneumon*	Family Herpestidae	*T*. *canis*	Worms
Asian palm civet	*Paradoxus hermaphroditus*	Family Viverridae	*Toxocara* spp.	Eggs
African civet	*Civettictis civetta*	Family Viverridae	*Toxocara* spp.	Eggs
Pine marten	*Martes martes*	Family Mustelidae	*T*. *cati*	Eggs
Stone marten/	*Martes foina*	Family Mustelidae	*Toxocara* spp.	Eggs
Beech marten				
American mink	*Neovision vison*	Family Mustelidae	*T*. *canis*	Eggs
Raccoon^a^	*Procyton lotor*	Family Procyonidae	*T*. *canis*	Worms

a No eggs detected so authors cannot confirm with certainty that the racoon is a definitive host of *T. canis*

It should be noted that there is some debate in the literature about the status of American mink and pine martens as definitive hosts of *Toxocara* spp. In a recent paper by Tull et al. (2022) the eggs of *T*. *canis* were detected in American mink and in contrast to the observations of Borecka et al. (2013) in pine martens. However, Kołodziej-Sobocińska et al. (2020) reported on the seroprevalence of *Toxocara* in American mink from Poland (21.7% seroprevalence among 1100 mink) and concluded that American mink may serve as paratenic hosts for *T*. *canis* in the wild. Tull et al. (2022) speculate that the unembryonated *T*. *canis* eggs that they detected in American mink and pine martens may, in fact, have been swallowed from a contaminated environment and passed via scats, hence such animals may be acting as transport hosts.

A recent experimental study by Klockiewicz et al. (2019a) revealed that American mink may act as paratenic hosts for *T*. *canis* and *T*. *leonina*. Tissue larvae were found in experimentally infected farmed American mink; histopathological examinations of parenchymal organs and striated muscles revealed lesions resembling those observed in other paratenic hosts due to toxocarosis (Klockiewicz et al., 2019b). In addition, this infection was confirmed by both ELISA and Western blot (Klockiewicz et al., 2019a).

No eggs were detected in the faeces of the wild boar (*Sus scrofa*) from Mexico but utilising ELISA, 20% of the sampled animals were found to be seropositive for *T*. *canis* (de la Rosa Arana et al., 2021). In contrast, *T*. *cati* larvae were detected in muscle samples from both wild boars and ostriches in Italy after enzymatic digestion and molecular confirmation (Michelutti et al., 2021). In a paper by Peng *et*. *al*. (2020), the authors describe the detection of *T*. *cati* eggs in 77.4% of scats collected from Amur tigers (*Panthera tigris altaica*) from China, and include a speculative route of transmission from wild boars, a preferred dietary item for these carnivores.

A recent survey of the helminths of the invasive raccoon (*Procyon lotor)* in Germany, revealed that a single animal from a sample of 102 free-ranging raccoons was infected with *T*. *canis* (prevalence 0.99%; Reinhardt et al., 2023). However, the authors did not detect eggs of *T*. *canis* in the faeces of the same animal and therefore could not confirm the raccoon's status as a final host for *T*. *canis* with certainty. However, these observations suggest that potentially raccoons could act as hosts for *T*. *canis* in the future.

## 6 Wild paratenic hosts: small mammals

Our knowledge of the significance of wild paratenic hosts, as sources of infection for definitive hosts, both domestic and wild, is still extremely limited and represents one of the largest gaps in our understanding of the epidemiology of *Toxocara* (Holland, 2017). Fecund adult *Toxocara* worms, inhabiting the intestinal tract of domestic and wild definitive hosts, release large numbers of eggs into the environment and these eggs can be consumed by paratenic hosts. Paratenic hosts can then act as sources of infection after predation by carnivorous definitive hosts and can aid such infective stages in avoiding unfavourable conditions, thereby bridging the gap in time during a temporary absence of definitive hosts. Our knowledge of the relative infective capacity of a range of vertebrate and invertebrate paratenic hosts is virtually non-existent (Holland and Hamilton, 2006). There are only a small number of published studies on the seroprevalence and larval burdens of *Toxocara* in wild paratenic hosts and most of these are confined to small mammalian hosts (Holland, 2017). Until relatively recently, none of those studies had identified the species of *Toxocara* larvae within the tissues of the paratenic hosts.

Despite being published over two decades ago, the study by Dubinsky et al. (1995) from Slovakia remains one of the most comprehensive with 11 small mammal species investigated for the presence of parasite-specific antibodies in sera and the presence of *Toxocara* larvae in the brain and the hind leg femoral muscles. Considerable variation between hosts was observed with no detectable seropositivity in *Rattus norvegicus* and the highest level recorded (32%) in the house mouse (*Mus musculus)*. The intensity of larval burdens was low (in the order of one–three larvae per brain) relative to the proportion of seropositive animals, with a higher intensity observed in mammals from suburban locations. Dubinsky et al. (1995) concluded that small mammals could act as important foci for the circulation and maintenance of *Toxocara* in the environment. More recently, Antolova et al. (2004) reported *Toxocara* seropositivity from 10 non-commensal rodent species from the Slovak Republic, confirming the higher seropositivity from suburban locations but identifying the highest seropositivity in *Apodemus agrarius* (21%). Among four commensal rodent species, Reperant et al. (2009) found the highest *Toxocara* seroprevalence (13·2%) in animals sampled from urban areas. The authors also highlighted that in parts of the world where raccoons are present, small mammals could act also as important sentinels for the presence of the highly pathogenic and emerging zoonotic infection, *Baylisascaris procyonis*. In N.E. Poland, Krupińska et al. (2023) investigated four rodent species from both grassland and forest habitats. They reported a 16-fold higher seroprevalence among grassland inhabiting species (*Microtus arvalis, Microtus agrestis*, *Alexandromys oeconomus*) (15.7%) in contrast to the forest dwelling *Myodes glareolus* (0.98%). It was concluded that the striking difference could be explained by higher contamination of grasslands by domestic dogs and wild canids, emphasising the important role of small mammals in perpetuating the transmission of *Toxocara*. In an important advance, Krucken et al. (2017) identified the species of *Toxocara* detected in 257 rodents of six different species with *Apodemus flavicollis* being the most common species, trapped in Berlin, Germany. A total of 8 *T*. *canis* (prevalence rate of 3.6%) and 4 *T*. *cati*-positive animals (prevalence rate of 1.6%) were identified by means of PCR, predominately in muscle. No *B*. *procyonis*-positive rodents were detected.

## 7 Environmental contamination

Although domestic dogs are likely to be the primary source of environmental contamination with *Toxocara* ova in urban environments (Airs et al., 2022; Panova and Khrustalev, 2018; Nijsse et al., 2015a), wildlife hosts and probably most importantly red foxes may also play a critical role due to their higher population densities in cities. However, a major challenge remains in understanding the relative contribution of domestic versus wild definitive hosts to environmental contamination with *Toxocara* spp. ova.

In an innovative approach, Morgan et al. (2013) developed an improved quantitative framework for the epidemiology of egg contamination in the city of Bristol, UK. A modelling approach, utilising a variety of empirical data (parasitology – prevalence, egg density and host data – faecal output, population density, age and status (owned vs stray)), led to the conclusion that in the absence of a large population of stray dogs and cats, pet dogs (especially those less than 12 weeks of age), dominate as the major source of total egg output into the environment. However, under certain circumstances, red foxes were found to play a role as contributors to egg contamination. Building upon the work of Morgan et al. (2013), Nijsse et al. (2015b) developed a stochastic model that included red foxes in addition to household dogs, household cats, stray cats (all older than 6 months of age) to environmental contamination with *Toxocara* spp. eggs in the Netherlands. Stray dogs were not included because such animals do not exist in the Netherlands. Both parasite variables (prevalence and abundance of infection) and host factors (density, coprophagic behaviour, faeces disposal by owners, cats’ outdoor access) were included in the model. Similar to the findings of Morgan et al. (2013), household dogs were found to be the main contributors to environmental contamination with *Toxocara* ova. However, stray cats, owned cats and red foxes also contribute eggs to the environment, and in urban areas egg output is dominated by stray cats. The authors made an important observation that due to the role of stray cats and red foxes (and stray dogs in other contexts), control measures that focus upon household pets alone are not sufficient to reduce environmental contamination to very low levels. Such modelling methodologies provide a framework for a more hypothesis-driven approach to the study of environmental contamination, a focus that remains sorely lacking (Holland, 2017).

In a recent study of *Toxocara* egg contamination of spinach sampled from four commercial farms in the south of England, (intriguingly entitled “from fox to fork”), Healy et al. (2023), detected 23% of the samples to be positive for *T*. *canis* and 1.7% for *T*. *cati*. In one farm, the prevalence of *T*. *canis* contamination was 45.5%. The authors concluded that given the preponderance of *T*. *canis* eggs detected, the source of contamination was most likely to be either dogs or red foxes, rather than cats. Given that spinach is often eaten raw, this enhances the likelihood of successful parasite transmission to humans.

The difficulty in making comparative observations between studies on environmental contamination was emphasised by Nijsse et al. (2020) and ascribed to the multiple factors that influence successful *Toxocara* egg detection. These include spatial variation and soil depth, method of detection (use of detergent, flotation method) and whether egg viability has been assessed. Many studies do not identify the *Toxocara* eggs recovered to species level. In an excellent long-term study in Poland, over a 20 year period, Mizgajska-Wiktor et al. (2017) found that *T*. *cati* eggs were much more commonly observed in urban areas compared to *T*. *canis* eggs, whereas the opposite was true for rural areas. The authors recommended that in order to be more effective, preventive measures should be preceded by discrimination of *T*. *canis* and *T*. *cati* eggs recovered from soil.

## 8 Transmission of *Toxocara* from companion animals to wild hosts?

The focus of this review has been on the population biology of *Toxocara* spp. infection in wild hosts and their potential to transmit infection to both companion animals and humans via environmental contamination. However, we must also consider the opposite situation i.e. transmission of *Toxocara* from companion animals to wild hosts. For obvious reasons, we are not yet in a position to unravel the complexity of such ecosystem interactions. However, if we give further consideration to small mammals that can act as sentinels of *Toxocara* egg contamination in their environment (see section 6 above) we can gain some insights. For example, in the work of Stuart et al. (2020), who sampled both woodmice (*Apodemus sylvaticus*) and voles (*Myodes glareolus*) from rural and urban sites in Ireland, larval cestodes were detected in both host species. Both woodmice and voles were infected with the larval stages of the cestodes *Taenia polyacantha* and *Taenia martes*. Woodmice were also infected with the larval stage of *Hydatigera taeniaeformis*. Mustelids and foxes can act as definitive hosts of *T*. *martes* whereas *H*. *taeniaeformis* is primarily a parasite of cats (but dogs can also act as definitive hosts) and *T*. *polycantha* is a parasite of canids, especially foxes. What these observations confirm is that eggs of these cestodes are shed by the definitive hosts whether they be companion or wild animals that contaminate the environment and can then be ingested by the small mammals that act as intermediate hosts. Ingestion of such hosts can then lead to adult worm infection in the appropriate definitive host. A similar process can occur with *Toxocara*, in that eggs from the environment whether they arise from companion or wild hosts, are ingested by these small mammals and larvae remain in the tissues. If predation occurs, adult worms can develop in the definitive host intestine. These observations highlight the potential interaction between parasites emanating from companion animals or wild animals that can both contaminate the environment and infect potential paratenic hosts.

Furthermore, the extent of *Toxocara* infection in companion animals varies considerably across the world as demonstrated in the recent reviews of Rostami et al. (2020a,b). For example, in the case of dogs, the estimated prevalence varied across WHO regions with the highest values of 19.2% in the Eastern Mediterranean region versus the lowest values of 6.4% in the Western Pacific. Rural dogs had a higher prevalence of infection as did regions at a low geographical latitude, close to the equator, characterized as having tropical climates. These observations suggest variability – across global regions and between urban and rural environments - in the likelihood of wild hosts being exposed to infection from companion animals.

## 9 Conclusion

This review has highlighted that an extensive number of wild carnivores are capable of acting as definitive and paratenic hosts of *Toxocara* spp. However, their roles as disseminators and reservoirs of infection has tended to be disregarded. As wildlife encroaches into urban areas or urban areas encroach into “the countryside” this is likely to contribute to an enhancement of their importance (Otranto and Deplazes, 2019). Ecology, diet and population size of the wildlife species clearly vary and will influence their relative zoonotic importance (Han et al., 2021). A key determinant of zoonotic disease emergence is the overlapping of environmental conditions with biological traits that enable parasites to be shared by humans and other animals (Wells et al., 2018).

At present, red foxes are the dominant species both in terms of the attention they receive in the published literature, but also the higher values of prevalence and abundance of *T*. *canis* reported. Furthermore, their increasing population densities and encroachment into urban areas enhance their potential to transmit *T*. *canis* to domestic dogs and humans via environmental contamination and their dietary preferences for a wide range of potential paratenic hosts contributes to increased exposure to infection. One study that illuminated the potential of the red fox to undermine successful control of *T*. *canis* in domestic dogs is the case of the red fox in Australia. Unlike in many other countries, the prevalence of *T*. *canis* in domestic dogs and contamination of the environment with *Toxocara* spp. ova is low (see Palmer et al.*,* 2008; Massetti et al., 2022) but the prevalence of *T*. *canis* in the red fox is higher (Dybing et al., 2013) and this highlights the potential of infection in foxes to spillover into both domestic animals and humans (Bezerra-Santos et al., 2023).

Other important external factors, such rising temperatures due to climate change, may also contribute to the epidemiology of *Toxocara* species eggs in the environment. In the Arctic, for example, climatic change may provide a better environment for red foxes, resulting in increasing population densities of the species that in turn contribute to spillover of infective parasite stages into local wildlife such as arctic foxes and wolves (Jenkins et al., 2013). *Toxocara* species eggs in soil may also survive better and retain infectivity for longer as temperatures increase, as discussed previously with respect to *Ascaris lumbricoides* by Blum and Hotez (2018).

In contrast, the population density of wolves is considerably less than that of red foxes and in many parts of the world, the wolf population remains fragmentary and in recovery after many years of persecution (Craig and Craig 2005, Lesniak et al., 2017). Therefore, the risk of pathogen spillover is likely to remain low. However, as discussed by Lesniak et al. (2017), wolves are a recolonising and an expanding apex predator in Central Europe including Germany. In their study, per capita endoparasite species richness and diversity significantly increased with population size and changed with age, whereas sex, microsatellite heterozygosity, and geographic origin had no effect. The authors conclude that their findings suggest that wolves from Central Europe currently constitute only a minor relevance as a reservoir of zoonotic parasites.

Most members of the family Felidae are solitary and territorial, in contrast to canids that tend to be more social (Riley et al., 2004). Therefore, although less well studied, based on current data wild felids are less likely to contribute significantly to pathogen spillover to domestic animals and humans. However, as outlined previously, prevalence and abundance of *T*. *cati* in such hosts is comparatively high indicating that their role as potential contributors to environmental contamination and parasite transmission cannot be discounted.

As emphasised by Reinhardt et al. (2023), exploring risk factors in final and intermediate wildlife hosts and the environment will provide a better understanding of the epidemiology of helminths, the role of wildlife, and the potential negative consequences of infectious pathogens including transmission of zoonotic parasites to humans.

## Declaration of competing interest

There are no conflicts of interest associated with this manuscript.
